# Development and validation of a nomogram model for predicting infection after radical resection of gastric cancer

**DOI:** 10.12669/pjms.41.5.11650

**Published:** 2025-05

**Authors:** Liang Zhou, Hong Wu, Xin Chen

**Affiliations:** 1 Liang Zhou, Department of General Surgery, BenQ Medical Center, Nanjing, Jiangsu Province 210019, P.R. China; 2 Hong Wu, Department of General Surgery, BenQ Medical Center, Nanjing, Jiangsu Province 210019, P.R. China; 3 Xin Chen, Department of General Surgery, BenQ Medical Center, Nanjing, Jiangsu Province 210019, P.R. China

**Keywords:** Gastric cancer, Infection, Nomogram model, Radical resection

## Abstract

**Objective:**

To develop and validate a nomogram model for predicting infection after radical resection of gastric cancer (GC).

**Methods:**

In this retrospective cohort study clinical data of patients who underwent radical resection of GC in BenQ Medical Center in Nanjing, China from January 2020 to April 2024 was retrospectively selected. Patients were randomly assigned to the training cohort and the validation cohort in a ratio of 7:3. The least absolute shrinkage and selection operator (LASSO) algorithm and logistic regression analysis were used to analyze the characteristics and screen the independent risk factors of infection after radical resection of GC to construct a predictive nomogram model. The prediction performance and clinical utility of the nomogram model were evaluated by drawing the receiver operating characteristic (ROC) and calculating the area under the curve (AUC), calibration curve, and decision curve analysis (DCA).

**Results:**

Records of 581 patients with GC after radical resection were included in this study. The incidence of postoperative infection was 19.1% (111/581). The nomogram model that included age, hypertension, open surgery, operation duration, lymphocyte count, and prognostic nutritional index (PNI) showed sufficient prediction accuracy, with the AUC of the training set and validation set of 0.833 (95% CI: 0.778-0.888) and 0.859 (0.859; 0.777-0.941), respectively. The calibration curve showed that the model’s predicted value is basically consistent with the actual value, and the calibration effect is good. DCA also shows that the predictive model has good clinical utility.

**Conclusions:**

The established nomogram model has a good predictive value in predicting infection after radical resection of GC in this study, which may be a reliable tool for clinicians to identify patients with GC at high risk of infection after radical gastrectomy.

## INTRODUCTION

Gastric cancer (GC) is a common malignant tumor of the digestive tract.^1^ According to the World Health Organization data, there were about 1032000 new diagnoses of GC worldwide in 2018 alone, with China accounting for about 42% of all cases.^1^-^3^ Furthermore, the incidence and mortality of GC in China are among the highest in the world.^1^-^3^ The treatment of GC varies according to the patient’s condition, age, physical condition, and other factors.^1^,^2^ Generally, early-stage GC can be cured by surgical resection, while advanced GC needs comprehensive treatment, including surgery, radiotherapy, chemotherapy, etc.^1^,^2^ Radical resection of GC is the first choice for the treatment of primary GC, and may effectively reduce the possibility of tumor recurrence and metastases.^4^,^5^

However, radical resection of GC is a relatively complex operation with a long postoperative recovery period,^4^ and complications, such as postoperative infection, bleeding, gastrointestinal dysfunction, and malnutrition.^5^,^6^ Recent studies have shown that postoperative infection is the main complication of radical GC resection.^7^,^8^ Since infection after radical resection of GC may also lead to other complications, such as sepsis and multiple organ dysfunction syndrome,^6^,^7^ early identification of patients at a high risk of postoperative infection is crucial to allow the selection of risk-adaptive surgery and perioperative interventions.^6^-^8^ While few predictive models for postoperative infection have been developed, they mainly consider all postoperative complications or only focus on pulmonary infections, and have not been validated with independent data.^9^-^11^

In this study, we aimed to develop and validate a nomogram model for predicting infection after radical resection of GC. This study randomly divided patients into a training and a validation set in a 7:3 ratio to construct and validate the prediction model. The results may provide a scientific assessment tool for high-risk patients and contribute to improving postoperative recovery and prognosis of GC patients.

## METHODS

This retrospective cohort study involved 581 patients who underwent radical gastrectomy in the Department of General Surgery at BenQ Medical Center in Nanjing, China, from January 2020 to April 2024.

### Ethical approval:

The ethics committee of our hospital approved this study with the number MJLL-2024125, Date: November 5, 2024.

### Inclusion criteria:

- Primary GC was diagnosed by postoperative pathological examination.
- Radical gastrectomy was performed.
- No other organ infection was found before the operation.
- The clinical data and postoperative follow-up data were complete.

### Exclusion criteria:

- Preoperative neo adjuvant and other methods of treatment.
- Tumors in other organs (such as liver cancer, lung cancer).
- Serious complications (such as cerebral infarction, renal failure, heart failure).
- Invasion of adjacent organs or distant metastases.

A total of 581 patients were selected according to their date of admission and designated as the whole queue data set. Using the “train_test_split” method in Python, the entire queue data set was randomly divided into the training and the verification cohorts at a ratio of 7:3. There were 407 patients in the training cohort and 174 patients in the validation cohort.

The patient’s age, gender, body mass index (BMI), history of smoking, drinking, hypertension, diabetes, coronary heart disease, chronic obstructive pulmonary disease, and previous abdominal surgery, American Society of Anesthesiologists physical status (ASA) classification, tumor diameter, differentiation, tumor TNM stage, operation method, surgical duration, intraoperative blood loss and other general clinical data were collected. The preoperative blood examination results were collected, including preoperative serum albumin, preoperative hemoglobin, preoperative neutrophil count, preoperative lymphocyte count, preoperative monocyte count, preoperative platelet count, preoperative carcinoembryonic antigen (CEA), preoperative carbohydrate antigen 199 (CA199) and other related indicators. The prognostic nutritional index (PNI) was calculated as follows: PNI=preoperative serum albumin (g/L)+5 × preoperative lymphocyte count (10^9^/L).

### Definition of postoperative infection:

Postoperative infections were defined as surgery-related infectious complications, including pulmonary infection, abdominal infection, incision infection, and urinary tract infection that occurred within 30 days after the operation.

### Statistical analysis:

All analyses were conducted using SPSS version 26.0 (IBM Corp, Armonk, NY, USA). Mann-Whitney U test was used to measure data with non-normal distributions, and a Chi-square test was used to analyze count data. Using the Lasso regression, combined with Lambda’s penalty coefficient, the regression coefficient of the variable was reduced to zero. Variables with zero regression coefficients were excluded, while those without zero regression coefficients were considered to be associated with postoperative infection. Multivariate logistic analysis was performed on the selected variables to determine the most relevant variables to form the basis for developing the nomogram model.

The validation cohort was used to evaluate the predictive ability of the developed nomogram model, including differentiation and calibration. Calibration curves were generated to illustrate potential differences between the training and validation cohorts, including the original and recalibrated nomogram models. The model’s discriminative ability was evaluated by receiver operating characteristic (ROC), using the area under the curve (AUC) as the measurement standard. The predictive ability of the final model was evaluated by comparing the observed incidence of postoperative infections. In addition, decision curve analysis (DCA) was conducted to evaluate the clinical application value of the model and calculate the net benefits under various risk threshold probabilities. R4.2.1 software was used for statistical analysis of predictive models. *P*<0.05 was considered statistically significant.

## RESULTS

This study included 581 eligible patients (316 males and 265 females) who underwent radical gastrectomy for GC and were randomly divided into a training cohort (n=407) and a validation cohort (n=174) at the 7:3 ratio. Most patients (54.4%) were male. The age range was 43-82 years, with a median age of 63 (57-70). Within 30 days after surgery, 111 patients developed postoperative infections, with an incidence rate of 19.1% (111/581). Among them, the total incidence of postoperative infection in the training cohort and validation cohort was 17.9% (73/407) and 21.8% (38/174), respectively (Table-I). The clinical characteristics of the patients are shown in Table-II.

**Table-I:** Postoperative infection rates in the training and validation cohorts.

Type of infection	Training cohort (n=407)	Validation cohort (n=174)
Lung	38(9.3)	22(12.6)
Abdominal cavity	7(1.7)	5(2.9)
Incision	14(3.4)	6(3.4)
Urinary system	6(1.5)	2(1.1)
Lung+Abdominal cavity	2(0.5)	1(0.6)
Lung+incision	6(1.5)	1(0.6)
Abdominal cavity+urinary system	0(0)	1(0.6)
Total incidence rate	73 (17.9)	38(21.8)

**Table-II:** Clinical characteristics of patients.

Characteristics	Training cohort (n=407)	Validation cohort (n=174)	χ^2^/Z	P
Male (yes), n (%)	219 (53.8)	97 (55.7)	0.185	0.667
Age (year), n (%)	63 (57-70)	64.5 (58-70)	-0.796	0.426
BMI (kg/m²), M(P25/P75)	23.1 (20.4-25.4)	23.4 (20.6-25.6)	-0.551	0.582
Smoking (yes), n (%)	182 (44.7)	66 (37.9)	2.295	0.130
Drinking alcohol (yes), n (%)	121 (29.7)	48 (27.6)	0.272	0.602
History of hypertension (yes), n (%)	88 (21.6)	44 (25.3)	0.933	0.334
History of diabetes (yes), n (%)	91 (22.4)	35 (20.1)	0.361	0.548
Coronary heart disease (yes), n (%)	43 (10.6)	23 (13.2)	0.852	0.356
Chronic obstructive pulmonary disease (Yes), n (%)	29 (7.1)	17 (9.8)	1.169	0.280
Previous history of abdominal surgery (yes), n (%)	45 (11.1)	15 (8.6)	0.781	0.377
Differentiation degree, n (%)			0.359	0.549
Medium to high differentiation	230 (56.5)	103 (59.2)		
Low differentiation	177 (43.5)	71 (40.8)		
TNM staging, n (%)			1.154	0.283
I-II	131 (32.2)	64 (36.8)		
III	276 (67.8)	110 (63.2)		
T staging, n (%)			0.600	0.439
T1+T2	143 (35.1)	67 (38.5)		
T3+T4	264 (64.9)	107 (61.5)		
ASA staging, n (%)			0.525	0.469
I+II	270 (66.3)	110 (63.2)		
III+IV	137 (33.7)	64(36.8)		
Surgical method, n (%)			0.314	0.575
Laparoscopic surgery	251(61.7)	103(59.2)		
Open surgery	156(38.3)	71(40.8)		
Surgical duration (hour), M (P25/P75)	4.8(4.4-5.6)	5.05(4.2-5.7)	-0.165	0.869
Intraoperative bleeding volume (mL), M (P25/P75)	205(168-258)	203.5(174-265)	-0.359	0.719
Tumor diameter (cm), M (P25/P75)	4.6 (4.2-5.3)	4.8(4.1-5.2)	-0.542	0.588
Neutrophil count (10^9^/L), M (P25/P75)	3.8(3.5-4.6)	3.65(3.4-4.5)	-1.869	0.062
Lymphocyte count (10^9^/L), M (P25/P75)	1.52(1.25-1.64)	1.48 (1.24-1.62)	-1.403	0.161
Mononuclear cell count (10^9^/L), M (P25/P75)	0.47 (0.44-0.54)	0.49 (0.43-0.58)	-1.129	0.259
Platelet count (10^9^/L), M (P25/P75)	225 (205-247)	230 (204-254)	-0.758	0.449
Hemoglobin level (g/L), M (P25/P75)	125 (106-147)	128 (109-154)	-1.109	0.267
CEA (ng/mL), M(P25/P75)	2.63 (2.14-2.95)	2.55 (1.9-3.21)	-0.880	0.379
CA199 (U/mL), M(P25/P75)	10.5 (8.4-13.5)	11.45 (8.0-14.5)	-0.700	0.484
Albumin level (g/L), M (P25/P75)	39 (36-42)	40 (34-43)	-0.451	0.652
PNI, M(P25/P75)	47 (43-50)	48 (42-52)	-1.376	0.169
Postoperative infection, n (%)	73 (17.9)	38 (21.8)	1.201	0.273

*Note:* BMI: body mass index; ASA: American Society of Anesthesiologists; CA: carcinoembryonic antigen; CA199: preoperative carbohydrate antigen; PNI: prognostic nutritional index.

**Fig.1:**
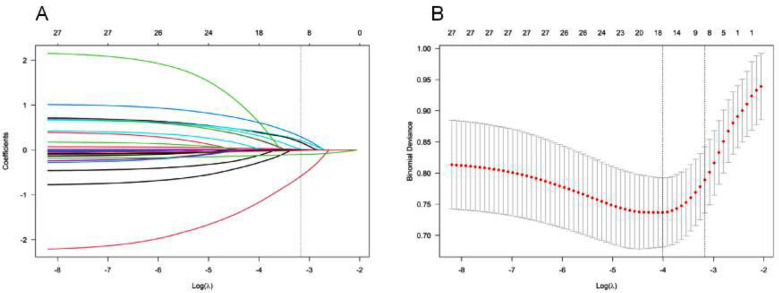
Lasso coefficient curve of infection after radical resection of GC. A: Each curve in the figure presents the change of each variable in coefficient. The ordinate is the coefficient value, the lower abscissa is log(λ), and the upper abscissa is the number of non-zero coefficients in the model at this time. B: 10-fold cross-cross validation fitting and then selecting the model

The Lasso regression algorithm was used for feature selection in the training cohort. This method helps to minimize the impact of multicollinearity and provides strong predictability and stability. Factors were selected based on the minimum partial likelihood binomial bias, and Lasso regression retained eight non-zero coefficient variables (Fig.1) that were considered significantly correlated with postoperative infection. The identified variables included age, alcohol consumption, hypertension, traditional open surgery, surgery time, lymphocyte count, albumin level, and prognostic nutritional index (PNI).

To further investigate the predictive significance of eight identified factors, they were next used for the multiple logistic regression analysis. The results showed that six variables, including age [odds ratio (OR)=1.045; 95% confidence interval (CI)=1.007-1.084; *P*=0.021], hypertension (OR=2.012; 95% CI=1.028-3.940; *P*=0.041), open surgery (OR=2.747; 95% CI=1.493-5.055; *P*=0.001), surgical duration (OR=1.741; 95% CI=1.245-2.433; *P*=0.001), lymphocyte count (OR=0.128; 95% CI=0.047-0.351; *P*<0.001), and PNI (OR=0.857; 95% CI=0.782-0.938; *P*=0.001), were independent risk factors for postoperative infection. The detailed results of multivariate logistic regression analysis are shown in Table-III.

**Table-III:** Analysis of risk factors for postoperative infection.

Independent variables	B	95% CI	P
Age	0.044	1.045(1.007-1.084)	0.021
Hypertension	0.699	2.012(1.028-3.940)	0.041
Open surgery	1.010	2.747(1.493-5.055)	0.001
Surgical duration	0.554	1.741(1.245-2.433)	0.001
Lymphocyte count	-2.056	0.128(0.047-0.351)	<0.001
PNI	-0.155	0.857(0.782-0.938)	0.001

*Note:* B is the regression coefficient; PNI: prognostic nutritional index.

**Fig.2:**
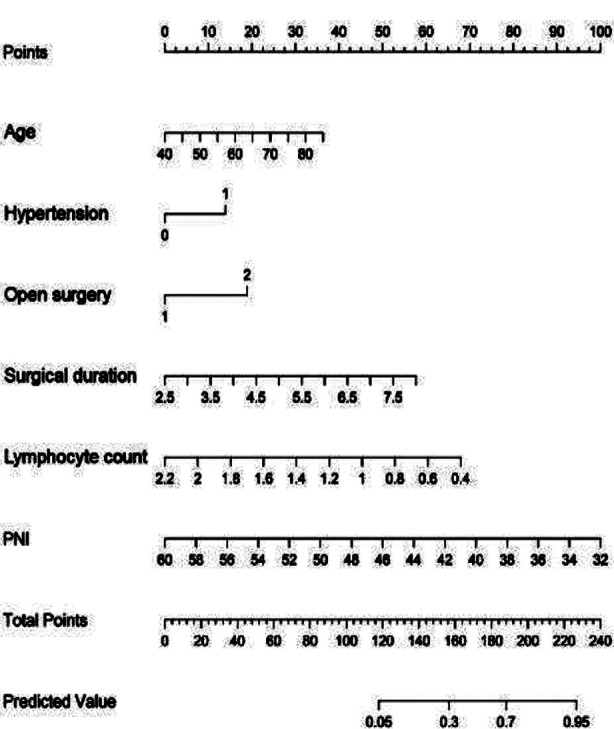
Prediction nomogram model for infection after radical resection of GC. Each level of the predictor variable represents a specific score. The total score is generated by summarizing the scores of each predictor variable. The total score corresponds to the probability of postoperative infection. GC: gastric cancer; PNI: prognostic nutritional index.

A Nomgram model for predicting the risk of postoperative infection was then constructed based on the six independent risk factors mentioned above (Fig.2). The corresponding score of each variable can be obtained by projecting to the top “points” axis according to the patient’s actual situation. In the same way, the total points are obtained by adding the corresponding scores of each variable. By projecting the total points to the bottom “Predicted value” axis, the infection after radical gastrectomy for GC can be estimated. For example, a 60 year old patient (16 points) with concomitant hypertension (14 points) underwent open surgery (19 points), surgery time was 5.5 hours (30 points), lymphocyte count was 1.0×10^9^/L (45 points), PNI was 48 (40 points), for a total of 164 points, which means the probability of predicting postoperative infection is approximately 38%. On the Hosmer Lemeshow test, the training cohort was *χ^2^*=8.427, *P*=0.393, and the internal validation cohort was *χ^2^*=24.193, *P*=0.189. This result indicates that the predicted results were close to the observed results. The ROC curve in the training cohort showed good discriminability (AUC: 0.833; 95% CI: 0.778-0.888).

**Fig.3:**
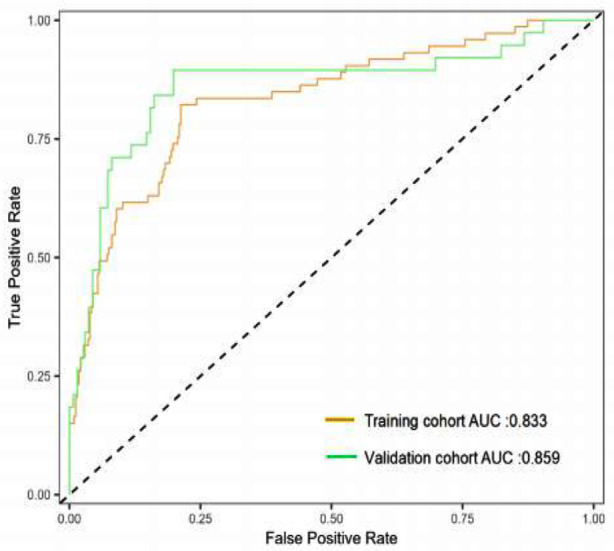
ROC curve and AUC of nomogram model. ROC: receiver operating characteristic; AUC: under the curve.

**Fig.4:**
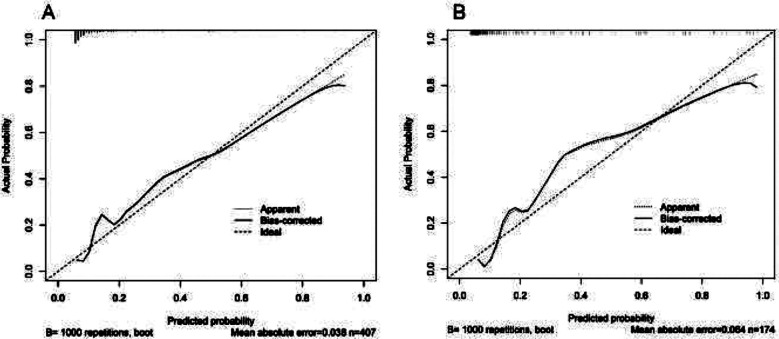
Calibration diagram of the prediction model. A. Calibration chart of the training cohort. B. Calibration chart in the internal validation cohort. The x-axis represents the predicted probability of infection after radical resection of GC. The y-axis represents the observed postoperative infection. The diagonal dashed line represents the perfect prediction of the ideal model. The solid line represents the performance of the nomogram model.

**Fig.5:**
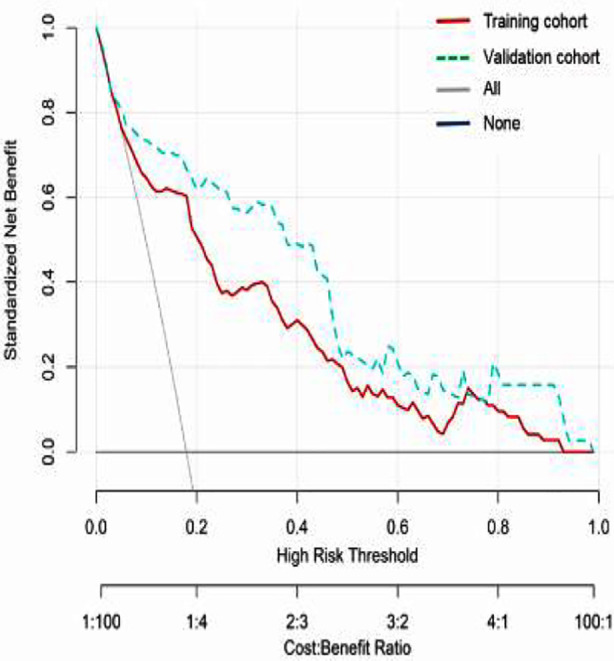
DCA of the nomogram model. The x-axis displays the threshold probability, while the y-axis measures the net benefit calculated by adding true positives and subtracting false positives. DCA: decision curve analysis.

The discriminative performance of the model was validated in the validation cohort (0.859; 0.777-0.941) (Fig-3). In addition, calibration curve analysis showed good consistency between predicted probabilities and observed postoperative infection rates in both the training and validation cohorts (Fig-4). DCA curves were drawn using training set data and validation set data separately (Fig-5). The DCA curves of the training and validation sets indicated that the prediction model has good clinical practicality.

## DISCUSSION

This study identified age, hypertension, open surgery, surgical duration, lymphocyte count, and PNI as independent risk factors for postoperative infection. The nomogram model generated using these factors demonstrated good predictive value and may help identify GC patients at high risk for postoperative infection. Of 581 patients in the cohort who underwent radical gastrectomy for GC, 111 had postoperative infections, with an incidence rate of 19.1% (111/581). Our results are consistent with previous reports that infection is the most common complication of radical gastrectomy for GC. In the study by Li et al.^12^, the incidence of infection after radical resection of GC was 23.3%. Another study by Liu et al.^10^ included 429 patients who underwent radical gastrectomy for GC, with a postoperative infection rate of 20.05%. In a meta-analysis of 32 studies, Chen et al.^13^ found that the incidence of infection after radical gastrectomy for GC ranged from 3.0% to 28.6%. Therefore, reducing the incidence of postoperative infection is a significant challenge clinicians face.

In this study, a simple, effective, and clinically useful model was developed to predict the likelihood of infection after radical resection of GC. This nomogram model combines simple risk factors, including age, hypertension, open surgery, surgery duration, lymphocyte count, and PNI. All variables are relatively easy to collect in clinical practice. In contrast to previous models that have mostly predicted the risk of infection based on the surgical sites or focused on lung infections,^9^-^13^ this model allows clinicians to calculate the risk of various postoperative infections, including lung, abdominal, incision, and urinary tract infections, promptly.

Therefore, the predictive model developed in this study can help with management decisions for postoperative infections. The results of this study indicate that age is one of the independent risk factors for infection after radical resection of GC. Elderly patients have weakened immune system function and poor resistance to pathogens.^14^ Moreover, the surgery may introduce the bacteria through incision and the use of instruments.^15^ Additionally, the tissue repair function of elderly patients is poor, and the wound healing time is long, increasing the risk of infection.^14^,^15^ Our results concur with previous observations that advanced age is one of the main influencing factors for postoperative infections.^15^,^16^

In agreement with previous research, this study identified hypertension as an independent risk factor for surgical site infections in GC patients.^16^-^18^ This effect of hypertension may be explained by the higher probability of vascular diseases, particularly arteriosclerosis and stenosis, in hypertensive patients.^17^,^18^ This may make the tissue more prone to ischemia and hypoxia during the surgical process, thereby increasing the risk of infection.^19^ This study also found that compared to laparoscopic surgery, the risk of postoperative infection in GC patients undergoing open surgery increased by 2.747 times. Additionally, the duration of surgery was also identified as a risk factor for infection after radical resection of GC. This is consistent with previous studies.^10^,^20^ Prolonged surgical duration and open surgery may lead to prolonged contact between the abdominal cavity and external pathogenic microorganisms, increasing intraoperative blood loss and physical trauma.^21^ Additionally, the long contracted state of blood vessels may exacerbate ischemia and hypoxia, leading to an increased risk of postoperative infection.^22^,^23^

In this study, low PNI levels were an independent risk factor for radical resection of GC. PNI is a comprehensive clinical scoring tool that combines albumin and lymphocyte levels to reflect the nutritional and immune status of cancer patients.^10^ Numerous studies have demonstrated that the decrease in lymphocyte counts reflects the reduced immune function and increased risk of infection in patients.^24^-^26^ The research results of Tatara et al.^25^ showed that lymphocyte depletion predicts poor prognosis for elderly GC patients after radical gastrectomy. The results of this study also indicate that preoperative lymphocyte depletion is one of the risk factors for postoperative infection. Our results are also consistent with the research results of Xiao et al.^26^

Multiple studies have focused on specific postoperative surgical site infection^27^, intraabdominal infection^28^, and pulmonary infection^29^ in patients with GC following radical gastrectomy, but one study focusing on predicting nonspecific postoperative infection is still scarce. Dong et al.^30^ constructed a nomogram model and the model can effectively identify patient at high risk of postoperative infection. Similarly, a nomogram model developed in this study integrated all six independent risk factors for predicting postoperative infection in GC radical resection, and the nomogram model also has good discriminability, with ROC values of 0.833 and 0.859 for the training and validation cohorts, respectively. The six factors, identified in the study, are easily measurable and routinely available in clinical practice. By applying individual clinical indicators of patients, a nomogram model allowed us to intuitively obtain scores for various influencing factors and calculate the total score to get the probability of postoperative infection and identify high-risk patients. This easy-to-use model may be, therefore, used to assess the risk of infection after radical gastrectomy for GC, identify high-risk populations as early as possible, and targeted preventive measures to improve postoperative recovery and prognosis of GC patients.

### Limitations:

Firstly, the medical data in this retrospective study were all from a single center with a small sample size, which may have a certain degree of selection bias. Secondly, some patients were excluded from this study due to the lack of complication registration and clinical pathological data. Thirdly, to validate the prediction model, the entire queue was randomly divided into a training cohort and an internal validation cohort in a 7:3 ratio. External validation was not conducted. Further studies are needed to validate the results using an external validation cohort. The repeatability and robustness of the nomogram model need to be validated in prospective multicenter studies with larger datasets.

## CONCLUSION

This study identified age, hypertension, open surgery, surgery duration, lymphocyte count, and PNI as risk factors for postoperative infection in GC patients undergoing radical resection. Based on these six risk factors, a well-performing nomogram risk prediction model was constructed. This model provides a reliable tool for clinicians to identify patients with GC at high risk of infection after radical gastrectomy, and to promote targeted intervention measures in a timely manner. However, further prospective multicenter studies with a larger sample size are needed to confirm the model’s predictive performance.

### Authors’ contributions:

**LZ:** Study design, literature search and manuscript writing.

**HW and XC:** Data collection, data analysis and interpretation. Critical analysis.

**LZ:** Manuscript revision and validation, critical review.

All authors have read, approved the final manuscript and are responsible for the integrity of the study.
